# Preferentially Disrupted Core Hubs Within the Default-Mode Network in Patients With End-Stage Renal Disease: A Resting-State Functional Magnetic Resonance Imaging Study

**DOI:** 10.3389/fneur.2020.01032

**Published:** 2020-11-05

**Authors:** Chi Ma, Fen Tian, Min-ge Ma, Hua-wei Su, Jian-cong Fan, Zhan-hui Li, Yan-de Ren

**Affiliations:** ^1^Department of Radiology, The Affiliated Hospital of Qingdao University, Qingdao, China; ^2^Department of Nephrology, The Affiliated Hospital of Qingdao University, Qingdao, China; ^3^College of Computer Science and Engineering, Shandong University of Science and Technology, Qingdao, China

**Keywords:** end-stage renal disease, resting-state functional magnetic resonance imaging, default-mode network, functional connectivity, cognitive impairment

## Abstract

Neuroimaging evidence implies that cognitive impairment in patients with end-stage renal disease (ESRD) is related to the disruption of the default-mode network (DMN). The DMN can be divided into three functionally independent subsystems, which include the cortical hub subsystem [consisting of the posterior cingulate cortex (PCC) and the anterior medial prefrontal cortex (aMPFC)], the dorsal medial prefrontal cortex (dMPFC) subsystem, and the medial temporal lobe (MTL) subsystem. However, it is unknown how the functional connectivity (FC) in DMN subsystems is differentially impaired in ESRD. This prospective study was carried out at the Affiliated Hospital of Qingdao University, China, between August 2018 and July 2020. Thirty-two ESRD patients and forty-five healthy controls (HCs) were recruited for this study and received resting-state functional magnetic resonance imaging (rs-fMRI) scanning, and FCs on predefined regions of interest (ROIs) were individually calculated in three DMN subsystems using both ROI- and seed-based FC analyses to examine FC alterations within and between DMN subsystems. The two-sample *t*-test was used for the comparisons between groups. We also tested the associations between FC changes and clinical information using Pearson's correlation analysis. The results demonstrated that ESRD patients, compared with HCs, exhibit reduced FC specifically within the cortical hubs and between the DMN hubs and two subsystems (the dMPFC and MTL subsystems). Moreover, the FC values between the aMPFC and PCC were positively correlated with creatinine and urea levels in the ESRD patients. Our results suggest that the cortical hubs (PCC and aMPFC) are preferentially disrupted and that other subsystems may be progressively damaged to a certain degree as the disease develops.

## Introduction

Individuals with end-stage renal disease (ESRD), defined as a glomerular filtration rate (GFR) of <60 ml min^−1^ 1.73 m^−2^ or a perpetual loss of >90% of normal renal function as the end stage of chronic kidney disease (CKD) (1), have a substantially higher prevalence of cognitive impairment than the general population; cognitive impairment is present in a striking 10 to 40% of ESRD patients, depending on the evaluation methods of cognitive impairment and the stage of chronic kidney disease (2). Cognitive impairment may affect independence, daily functioning, and medication adherence of the patients (3) and is also an independent predictor of mortality (4). Hemodialysis, an irreplaceable treatment for ESRD, may lead to rapid fluid transfer and swings in blood pressure. Hemodynamic instability may ultimately result in brain damage (5). Small-vessel cerebrovascular disease, uremic metabolite accumulation, and anemia may also be important factors in the development of CKD-related cognitive impairment (6). Moreover, long-term hemodialysis may result in significantly reduced quality of life, contributing to the development of anxiety and depression. It should be noted that even younger ESRD patients have poorer cognitive function than their peers (7). Therefore, what are the neuropathological mechanisms of cognitive dysfunction in ESRD patients? It would be worthwhile to use a neuroimaging technique to precisely determine how cognitive impairment affects the brain, as it may provide a sensitive neurobiological signature that would enable earlier, accurate clinical diagnosis and allow an effective therapeutic intervention.

In fact, the connectivity between the brain may be interrupted in accordance with this type of brain injury. Previous diffusion tensor imaging (DTI) (8) and voxel-based morphometry (VBM) (9, 10) studies have shown local impairment of white matter integrity and decreased gray matter volume. Resting-state functional magnetic resonance imaging (rs-fMRI) research has also demonstrated that functional connectivity (FC) within and among several different cortical networks is altered in ESRD (11, 12). By detecting the correlations of intrinsic fluctuations in blood oxygenation level-dependent (BOLD) signals among different regions, FC analysis, a common method of resting-state functional magnetic resonance, can sensitively reflect the coordination and interaction of neural activities among different functionally related brain regions (13), thus providing us with a promising viewpoint for the early detection of brain injury in ESRD patients. Several resting-state networks [such as default mode network (DMN), salience network, executive network, and sensory motor network] have been identified consistently. Among the networks, the DMN, consisting of the posterior cingulate cortex (PCC), the precuneus cortex (Pcu), the prefrontal cortex, the lateral parietal cortex, the medial temporal lobe, and the hippocampus (14), has been paid increasing amounts of attention in the research of neuropsychological diseases related to cognitive impairment (15). The DMN is involved in self-referential cognition process. Previous behavioral studies have demonstrated that ESRD patients have multidomain DMN-related neurocognitive impairment, and disrupted connectivity in the DMN may affect a variety of cognitive processes in those patients, including concentration, executive, memory, abstraction, and judgment function (16), which may be the underlying neuropathological mechanism of ESRD cognitive impairment.

Recent studies have shown that the DMN functional alterations induced by ESRD are not limited to regional homogeneity (ReHo) (17) and amplitude of low-frequency fluctuation (ALFF) (18) changes; they are also manifested as impaired FC in independent component analysis (ICA) (19), seed-based FC analysis (20, 21), and graph theory-based analysis (22, 23). Most of these functional abnormalities involve the parietal lobe, PCC, Pcu, and medial frontal lobe. However, a detailed analysis of region-specific disconnections in the DMN has not been made available until now. Meanwhile, Andrews-Hanna et al. (24) found that the functional architecture of the DMN comprises three functionally separable subsystems: the core hub subsystem [consisting of the PCC and anterior medial prefrontal cortex (aMPFC)], the dorsal medial prefrontal cortex (dMPFC) subsystem [consisting of the (dMPFC), temporal parietal junction (TPJ), lateral temporal cortex (LTC), and temporal pole (TempP)], and the medial temporal lobe (MTL) subsystem [including the ventral MPFC (vMPFC), posterior inferior parietal lobule (pIPL), retrosplenial cortex (Rsp), parahippocampal cortex (PHC), and hippocampal formation (HF)]. Different subsystems in DMN cooperate with each other and participate in different cognitive processes. Therefore, studies on the altered interactions within and among the three subsystems of the DMN may provide new insights into the neuropathological mechanisms of various brain disorders. Efforts have been made in several clinical populations, such as people with Alzheimer's disease (25) and people with schizophrenia (26). These observations suggest that the patterns of impairment in the three subnetworks in these patients seem to differ. Thus, whether there is a special impaired pattern of DMN to distinguish ESRD-related cognitive impairment from other mental disorders is another interesting question that is worthy of further study.

To fill this knowledge gap, predefined 11 ROIs according to the research of Andrews-Hanna et al. (24) were used for FC analysis using both ROI- and seed-based FC analyses in this study. Regarding the distinction between the two methods, seed-based connectivity looks primarily at connections between a seed and all voxels in the brain. ROI-based connectivity observes connections between the regions of interest (13). Different analytical approaches could influence the results for DMN connectivity. Given that ROI-based analysis results rely on *a priori* ROIs to a large extent that may potentially affect the accuracy of the reproducibility measures, the advantage is the ability to directly answer questions about connectivity. Therefore, seed-based FC analysis was also performed to comprehensively verify and interpret the findings from ROI-based analysis.

The goals of the current study were as follows: (a) We sought to examine the abnormalities in interactions within and between DMN subsystems to provide further evidence of an aberrant DMN and to determine the impaired pattern of DMN in ESRD patients. (b) We attempted to determine whether there were associations between these FC changes and the clinical variables in patients with ESRD.

## Materials and Methods

### Subjects

The study was approved by the Medical Research Ethics Committee of the Affiliated Hospital of Qingdao University, China, and written informed consent was obtained from all subjects before the study. For this hospital-based prospective case–control study, 45 ESRD patients who were diagnosed with renal failure, defined by a GFR <15 ml min^−1^ 1.73 m^−2^, and who underwent regular hemodialysis were recruited from the nephrology and renal transplantation department at our hospital between August 2018 and July 2020. Concurrently, 45 healthy, age-, and gender-matched volunteers were recruited from the local community. To avoid possible coupling effects, all subjects in the present study were right-handed and younger than 60 years old. The demographic and clinical data of each ESRD patient were acquired from the electronic medical records in our hospital. All ESRD patients completed laboratory examination within 24 h before MR imaging, which included serum creatinine level, urea level, hemoglobin level, hematocrit level, cholesterol level, serum potassium, serum sodium, and serum calcium.

The shared exclusion criteria for patients and control subjects were as follows: (a) history of severe head injury or obvious brain lesions on T2-fluid-attenuated inversion recovery (FLAIR) images; (b) neurodegenerative diseases (e.g., epilepsy, Parkinson's disease, Alzheimer's disease); (c) acute cerebrovascular disease or peripheral arterial occlusion; (d) chronic liver failure or heart failure; (e) history of psychiatric disorders in any control subject or history of major psychiatric disorders in any subject; (f) severe metabolic diseases (e.g., primary hyperparathyroidism, diabetes); (g) substance abuse, including drugs, alcohol, or cigarettes; (h) pregnancy or lactation at the time of the study; and (i) contraindications to MRI and excessive head movement during the scan. Five ESRD patients were excluded due to lacunar infarct lesions. Eight patients were excluded during functional MR image preprocessing. The final study population included 32 patients with ESRD and 45 healthy controls (HCs). Details regarding the clinical and demographic data of the remaining subjects are shown in Table 1.

**Table 1:** Demographic and Clinical Data of the Two Study Groups.

**Variable**	**ESRD patients**	**Healthy controls**	***p*-value**
	**(*n* = 32)**	**(*n* = 45)**	
Age (years)	44.4 ± 15.0	38.7 ± 13.1	0.080^b^
Sex (male/female)	17/15	23/22	0.862^a^
Education (years)	12.3 ± 2.7	12.9 ± 3.4	0.404^b^
Dialysis duration (month)	12.3 ± 12.2	–	–
creatinine (μmol/L)	642.3 ± 332.6	–	–
Urea (mmol/L)	22.0 ± 9.0	–	–
Ca^2+^ (mmol/L)	2.1 ± 0.3	–	–
K^+^ (mmol/L)	4.5 ± 0.8	–	–
Na^+^ (mmo/L)	139.3 ± 3.2	–	–
Hemoglobin (g/L)	92.7 ± 18.9	–	–
Hematocrit	29.2 ± 6.0	–	–
Cholesterol (mmol/L)	4.8 ± 2.2	–	–

*Values are represented as the mean ± SD*.

*ESRD, end-stage renal disease*.

a *The p-value was obtained by Chi-square test*.

b *The p-value was obtained by two-sided two-sample t-test*.

### MR Data Acquisition

All MRI images were collected using a GE 3T MRI scanner (GE Medical Systems, Milwaukee, WI) equipped with a standard head coil. Participants were asked to remain awake, relaxed, keep their eyes closed, and not to do any specific thinking during functional data collection. Conventional imaging sequences, which included T1-weighted images and T2-FLAIR images, were acquired for each subject to detect clinically asymptomatic lesions.

Using an echo-planar imaging (EPI) sequence, the rs-fMRI data were obtained with the following parameters: repetition time (TR)/echo time (TE) = 3,000 ms/40 ms, flip angle = 90°, 25 slices, thickness/gap = 5/0 mm, matrix size = 96 × 96, and field of view = 24 × 24 cm, 128 time points. Each scan lasted ~6 min.

Three-dimensional brain high-resolution T1-weighted structural images were acquired using a 3D magnetization-prepared rapid-acquisition gradient-echo (3D-MPRAGE sequence). The parameters are as follows: 176 sagittal slices, TR = 5.6 ms, TE = 1.7 ms, matrix = 256 × 256, (FOV) = 25.6 × 25.6 cm, and thickness/gap = 1.2/0 mm. This session lasted for ~5 min.

### Data Preprocessing

The rs-fMRI data were preprocessed by the Data Processing Assistant for Resting-State fMRI (DPARSF) software (27) (http://www.restfmri.net) with the following steps: (a) Image format conversion (DICOM to NIFTI) and then removal of the first 10 time points to stabilize the longitudinal magnetization and to accustom the subjects to the rs-fMRI scan noise. One healthy subject was excluded due to format conversion errors. (b) Slice timing was performed to avoid the time phase difference between different slices. (c) The subjects who had excessive head motion were excluded, and the thresholds were set to mean framewise displacement (FD) per Jenkinson (28) <0.2 mm, head translation <3 mm, and rotation less than 3° in any direction. Eight patients were excluded due to excessive head motion. The mean FD values of the remaining participants of the two groups were (0.073 ± 0.039) and (0.061 ± 0.032), respectively. There was no significant difference in FD values between the two groups by the two-sample *t*-test (*t* = 1.557, *p* = 0.124). (d) The individual T1-weighted structural images were coregistered to functional images; segmented into gray matter, white matter, and cerebrospinal fluid (29); and spatially normalized to Montreal Neurologic Institute (MNI) standard space (resampling voxel size = 3 × 3 × 3 mm^3^). (e) Covariates, including head motion, white matter signal, and cerebrospinal fluid signal, were regressed out from the time series of each voxel. We used the Friston 24-parameter model (6 motion parameters, 6 temporal derivatives, and the 12 corresponding squared items) to regress out head motion effects (30). (f) Linear detrending and bandpass filtering (0. 01 < *f* < 0.08 Hz) were performed to reduce low-frequency drift and high-frequency cardiac or respiratory noise. (g) The images were smoothed with an isotropic Gaussian kernel with a full width at half-maximum of 4 mm. Then, FC was calculated based on the preprocessed fMRI data.

### FC Analysis

In our study, both ROI- and seed-based FC analyses were performed to investigate the abnormal FC within the DMN.

#### ROI-Based FC Analysis

**Figure 1:**
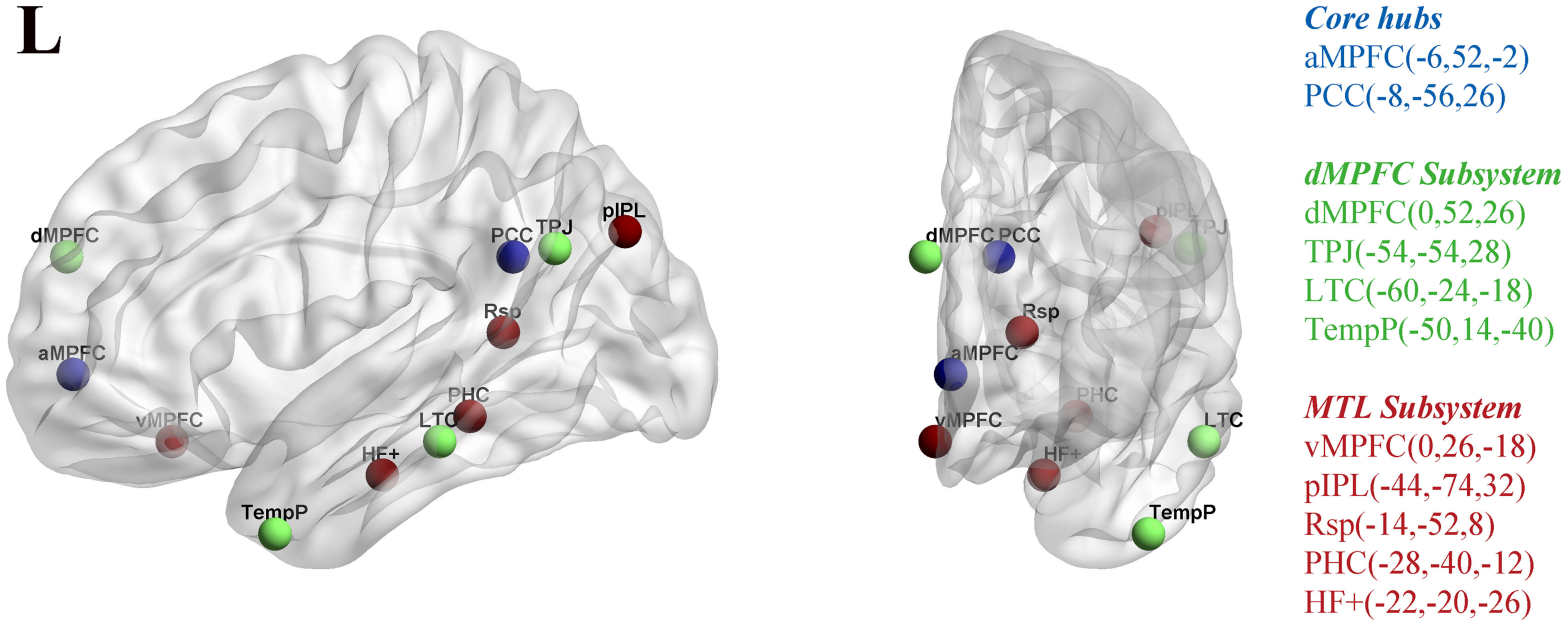
Eleven ROIs utilized in the rs-fMRI analysis for the midline core (blue), dorsal medial prefrontal cortex (dMPFC) subsystem (green), and the medial temporal lobe (MTL) subsystem (red) within DMN. The corresponding MNI coordinates for each ROI are shown on the right side of the picture. The regions were selected from a previous study (24). (ROI, regions of interest; DMN, default-mode network; PCC, posterior cingulate cortex; aMPFC, anterior medial prefrontal cortex; dMPFC, dorsal medial prefrontal cortex; TPJ, left temporo-parietal junction; LTC, lateral temporal cortex; TempP, temporal pole; MTL, medial temporal lobe; vMPFC, ventral medial prefrontal cortex; pIPL, posterior inferior parietal lobule; Rsp, retrosplenial cortex; PHC, parahippocampal cortex; HF, hippocampal formation).

To investigate the altered function and interaction of DMN subsystems, we performed a ROI-based FC analysis in a pairwise manner. Eleven ROIs of the DMN were approximated as spheres, each with a 5-mm radius (see Figure 1), according to the definition in a previous study (24). To reveal the functional connection patterns between these regions, we separately constructed their pairwise connectivity matrix using FC analysis. First, the temporal correlations between averaged time courses for each pair of ROIs were calculated and then transformed to *z* values using DPARSF software. Specifically, for each ROI, the average time series was calculated for each subject and then correlated with the time series of the other 10 ROIs. An 11 × 11 FC matrix was obtained for each subject. Next, a network-based statistics (NBS) method (31) was used to identify edges with significantly different FC in the ESRD patients compared with the HCs using the GRETNA software toolbox (32). Independently corrected *p*-values (after 10,000 permutations) were computed for each link using a generic procedure to control the familywise error rate (FWE) (33). A value of *p* < 0.05 was set as the threshold for significant differences in the present study. Age, gender, education, and head motion parameters (FD according to Jenkinson) were controlled for covariates in the between-group analyses.

#### Seed-Based FC Analysis

To comprehensively verify and supplement the findings from ROI-based analysis, four spherical ROIs (radius = 5 mm), selected on the basis of previous research (24), and centered at the PCC (−8, −56, 26), aMPFC (−6, 52, −2), dMPFC (0, 52, 26), and vMPFC (0, 26, −18), were generated to calculate seed-based FC in the current study. These four seeds were selected because they represent the cores of the three subsystems of DMN (24). For each seed, we used DPARSF software to extract the average time course. Then, the correlation between the averaged time course of each seed and the time series of the whole brain was computed in a voxelwise manner. Finally, we used Fisher's *r*-to-*z* transformation to transform the correlation coefficients into *z* values to improve the normality of their distribution. Two-sample *t*-tests were performed on the PCC/aMPFC/dMPFC/vMPFC-seeded FC maps of the two groups individually to identify regions with significant group differences [Gaussian random field correction with voxel-level threshold *p* < 0.001 and cluster-level threshold *p* < 0.01 (0.05/4), two-tailed] with age, gender, education, and head motion parameters (FD according to Jenkinson) as covariates.

### Statistical Analysis

#### Group Differences in Demographic and Clinical Data

The demographic and clinical data differences between the two groups were compared using the two-sample *t*-test and χ^2^ tests in SPSS 22.0 software (SPSS Inc., Chicago, IL, United States). Statistical significance was set to *p* < 0.05.

#### FC and Correlation Analyses

In the FC analysis, the regions showing significantly different FC between the ESRD and HCs were mapped to cortex surface and visualized with the BrainNet Viewer package (34). To investigate the potential effect of laboratory results on the DMN, we extracted the mean FC correlation coefficient values (after Fisher's *r*-to-*z* transformation) of these abnormal regions using DPABSF software. Then, we conducted a Pearson's correlation analysis between altered FC and clinical variables (dialysis duration, creatinine level, urea level, hemoglobin level, hematocrit level, cholesterol level, serum potassium, serum sodium, and serum calcium) in the ESRD patients.

## Results

### Participants' Demographic and Clinical Information

The demographic and clinical information of all patients and healthy subjects are shown in Table 1. There were no significant differences in age, gender, or education level between the two groups (*p* > 0.05).

### ROI-Based FC Result

**Figure 2:**
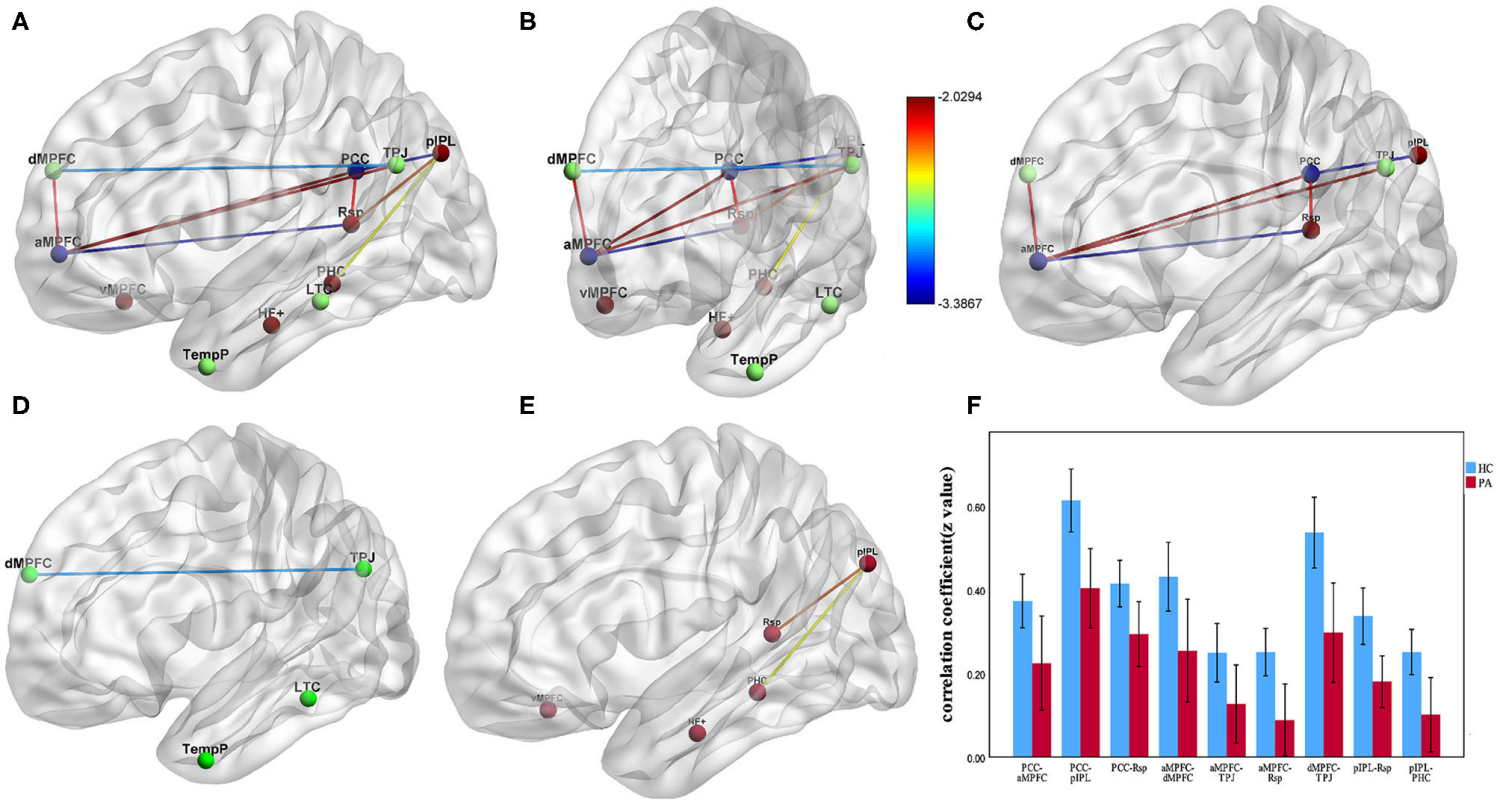
The group differences of pairwise FC between ESRD patients and HCs (*p* < 0.05, NBS correction). **(A–E)** Impaired FC in the regions of three DMN subsystems in ESRD patients compared with HCs. **(C)** Significantly decreased FC between the DMN hubs and two subsystems (PCC-aMPFC, PCC-pIPL, PCC-Rsp, aMPFC-dMPFC, aMPFC-TPJ, aMPFC-Rsp). **(D)** Significantly decreased FC between dMPFC and TPJ in the dMPFC subsystem. **(E)** Significantly decreased FC between pIPL and Rsp, pIPL, and PHC in the MTL subsystem. **(F)** Bar diagrams of average FC (Fisher's *r*-to-*z* transformed values) between the DMN core hubs and regions of the dMPFC and MTL subsystem. Error bars depict standard errors of the mean. Lines represent FC between each pair of defined ROI regions with significant group differences between two groups. The color bars and the color of lines represent the T scores. (FC, functional connectivity; ESRD, end-stage renal disease; HCs, healthy controls; NBS, network-based statistic; PCC, posterior cingulate cortex; aMPFC, anterior medial prefrontal cortex; dMPFC, dorsal medial prefrontal cortex; TPJ, temporal parietal junction; pIPL, posterior inferior parietal lobule; Rsp, retrosplenial cortex; PHC, parahippocampal cortex).

The ROI-based FC strength of the ESRD and HC controls is shown in Table 2 and Figure 2. In the dMPFC subsystem, compared with the controls, ESRD patients exhibited significantly reduced connectivity in the dMPFC-TPJ ROI pair. In the MTL subsystem, significantly reduced connectivity was found in the pIPL-Rsp and pIPL-PHC ROI pair. Interestingly, the ESRD patients exhibited hypoconnectivity between the DMN hubs and two subsystems (PCC-aMPFC, PCC-pIPL, PCC-Rsp, aMPFC-dMPFC, aMPFC-TPJ, aMPFC-Rsp), suggesting widespread functional disconnection between the DMN hubs and those two subsystems.

**Table 2:** Regions showing significantly decreased pairwise FC in patients with ESRD compared with HCs.

**ROI region**	**Connected brain ROI region**	***t*-value**
ESRD vs. HCs		
NBS correction (*p* < 0.05)		
PCC	aMPFC	−2.029
	pIPL	−3.040
	Rsp	−2.205
aMPFC	dMPFC	−2.191
	TPJ	−2.058
	Rsp	−3.387
dMPFC	TPJ	−3.040
pIPL	Rsp	−2.329
	PHC	−2.559

*FC, functional connectivity; ESRD, end-stage renal disease; HCs, healthy controls; NBS, network-based statistic; PCC, posterior cingulate cortex; aMPFC, anterior medial prefrontal cortex; dMPFC, dorsal medial prefrontal cortex; TPJ, temporal parietal junction; Rsp, retrosplenial cortex; pIPL, posterior inferior parietal lobule; PHC, parahippocampal cortex*.

### Seed-Based FC Result

**Figure 3:**
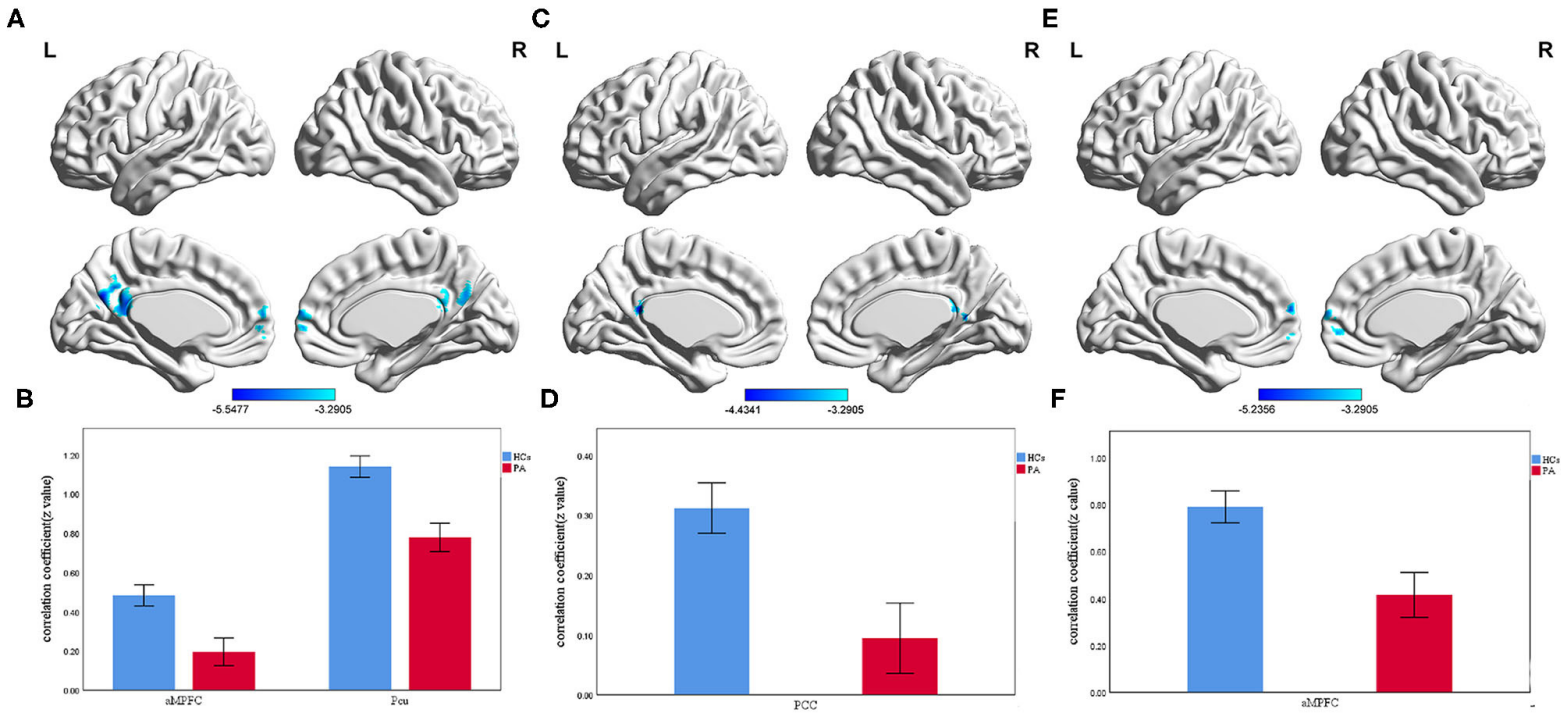
The group differences of voxelwise FC between ESRD patients and HCs (voxel-level threshold *p* < 0.001 and cluster-level threshold *p* < 0.01, GRF correction). **(A)** Significantly decreased PCC-seeded FC with bilateral aMPFC and Pcu in ESRD patients compared with HCs. **(B)** Bar diagrams of average PCC-seeded FC (Fisher's *r*-to-*z* transformed values) in the two groups. **(C)** Significantly decreased aMPFC-seeded FC with bilateral PCC in ESRD patients compared with HCs. **(D)** Bar diagrams of average aMPFC-seeded FC (Fisher's *r*-to-*z* transformed values) in the two groups. **(E)** Significantly decreased dMPFC-seeded FC with bilateral aMPFC in ESRD patients compared with HCs. **(F)** Bar diagrams of average dMPFC-seeded FC (Fisher's *r*-to-*z* transformed values) in the two groups. The color bars represent the T scores. The results were mapped onto the brain surface using the BrainNet viewer software. Error bars depict the standard error of the mean. (FC, functional connectivity; ESRD, end-stage renal disease; HCs, healthy controls; GRF, Gaussian random field; PCC, posterior cingulate cortex; aMPFC, anterior medial prefrontal cortex; Pcu, precuneus; dMPFC, dorsal medial prefrontal cortex).

Compared with HCs, ESRD patients exhibited significantly decreased PCC-seeded FC with the bilateral aMPFC and Pcu. For the aMPFC seed, only the bilateral PCC showed significantly decreased FC in patients with ESRD compared with HCs. For the dMPFC seed, the bilateral aMPFC showed significantly decreased FC in patients with ESRD compared with HCs (Table 3; Figure 3). However, there were no significant group differences in connectivity between the vMPFC seed and any region.

**Table 3:** Regions showing significantly decreased voxelwise FC in patients with ESRD compared with HCs.

**ROI region**	**Connected brain region**	**Side**	**Brodmann area**	**Cluster size**	**MNI coordinates (mm)**			***t*-value**
					**X**	**Y**	**Z**	
**ESRD vs. HCs (GRF correction, voxel-level threshold** ***p*** **<** **0.001, cluster-level threshold** ***p*** **<** **0.01, 2-tailed)**								
PCC	aMPFC	L/R	10	110	0	63	15	−5.1763
	Pcu	L/R	23	129	−6	−63	24	−5.5477
aMPFC	PCC	L/R	30	92	−6	−42	15	−4.4341
dMPFC	aMPFC	L/R	10	86	0	60	15	−5.2356

*FC, functional connectivity; ESRD, end-stage renal disease; HCs, healthy controls; MNI, Montreal Neurological Institute; GRF, Gaussian random field; PCC, posterior cingulate cortex; aMPFC, anterior medial prefrontal cortex; Pcu, precuneus*.

### Correlation Between FC and Clinical Variables

**Figure 4:**
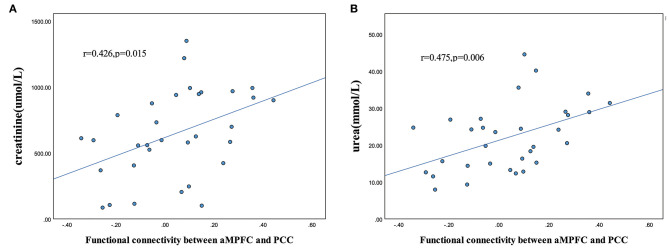
Scatter plot of the relationship between altered functional connectivity and clinical variables. Significantly positive correlations between aMPFC-PCC functional connectivity and creatinine level **(A)** and urea level **(B)** in the patients with ESRD (*p* < 0.05). (PCC, posterior cingulate cortex; aMPFC, anterior medial prefrontal cortex; ESRD, end-stage renal disease.)

For ESRD patients, FC values between the aMPFC and PCC extracted from the seed-based analysis were positively correlated with the creatinine level (*r* = 0.426, *p* = 0.015) and urea level (*r* = 0.475, *p* = 0.006) (Figure 4).

## Discussion

To date, this study is the first to focus on the neural activity of DMN subsystems in ESRD patients, although a previous work has investigated other aspects of ESRD. We report two major findings. First, the extensive functional disconnection within the DMN in ESRD patients was mainly between the DMN hubs and two subsystems. Second, the dMPFC and MTL subsystems in the DMN were also disrupted to a certain degree.

### Disrupted Cortical Hubs in Patients With ESRD

Our ROI-based FC analysis shows a widespread functional disconnection between the DMN hubs and those two subsystems (PCC-aMPFC, PCC-pIPL, PCC-Rsp, aMPFC-dMPFC, aMPFC-TPJ, aMPFC-Rsp). In addition, seed-based FC analysis shows significantly decreased FCs between the PCC and Pcu, the PCC and aMPFC, and the aMPFC and dMPFC in patients with ESRD compared with HCs, which were consistent with the results of ROI-based analysis. In line with our findings, Ni et al. (19) found through ICA that ESRD patients showed decreased FC in the PCC, precuneus, and MPFC, with further reduction in the MPFC with the development of minimal nephrotic encephalopathy. Similarly, Lu et al. (35) reported that significantly decreased FC of the DMN was observed in the PCC and Pcu, as well as in the MPFC, in the ESRD group with mild cognitive impairment. However, a previous study (21) using the PCC and vMPFC as seeds found that some other regions also had decreased FC with the PCC or vMPFC, such as the thalamus, middle temporal gyrus, and anterior cingulate gyrus, which was inconsistent with our results. The reasons for this difference may include the different sample sizes and the coordinates of selected seed points. As consistently reported in the literature, the aMPFC and PCC, as the midline cores of DMN, are strongly correlated with the dMPFC and MTL subsystems and share common functions, showing preferential autocorrelation activities in functional integration in all time contexts; they are activated when people make self-relevant affective decisions (24). Separately, the PCC has a central role in supporting internally directed cognition, retrieving autobiographical memories, planning for the future, and regulating attention (36). The aMPFC is involved in parts of cognitive and task performance and emotional response, including attention-demanding processes, evaluative judgment, self-referential processes, self-initiated thoughts, or emotional and intention processing (37). The DMN subsystems interact in a dynamic equilibrium, which is crucial for the maintenance of normal cognition, and the PCC and aMPFC may receive information integration from the other two subsystems. Thus, the widespread disruption in the functional integration of core hubs in DMN may be the main reasons for multidomain DMN-related cognitive dysfunction involving concentration, executive, memory, emotion, and judgment in ESRD subjects. In addition, a brain-network-based analysis also suggests that the nodal efficiencies of default-mode components were disproportionately weakened and tended to preferentially affect central or hub-like regions in ESRD patients (22).

Combined with the above findings, we proposed that cortical hubs (the PCC and aMPFC) in the DMN were preferentially disrupted; this is a specific pattern distinct from what is observed in other diseases; for example, in Alzheimer's disease (AD), disruption has been found mainly in the MTL subsystem (25). This interference can be explained and supported by a large number of previous studies. Evidence from previous DTI (8) and VBM (9, 10) studies of ESRD patients found that white matter damage and gray matter volume reduction are mainly located in the anterior frontal lobe. An MRS study by Wang et al. (38) also found cerebral metabolism changes (lower NAA/Cr ratio and higher Cho/Cr ratio) in bilateral prefrontal in ESRD patients. The prefrontal cortex plays a crucial role in cognitive control, including working memory, learning, and attention, as well as emotional response (39); even cognitively normal elderly individuals with white matter hyperintensities (WMHs) have been reported to have gray matter (GM) volume loss predominantly in the frontal cortex (40). Furthermore, the anatomical location of the PCC is located at the margin between the territories of the two main arteries supplying blood to the brain; therefore, it is susceptible to hemodynamic changes brought about by long-term hemodialysis (41). The PCC is one of the brain regions with the highest levels of metabolic activity and connectivity (42, 43). Using dynamic causal modeling, Davey et al. found that self-referential cognitive processes were driven by PCC activity and moderated by the regulatory influences of MPFC (44). Therefore, when considered with our current findings, this evidence indicates that the extensively disrupted FC between core DMN hubs and other subsystems in ESRD patients may be due to the weakened driving role of PCC. In our study, creatinine and urea levels in patients with ESRD were positively correlated with the *z*-values of FC between the aMPFC and PCC. This is a surprising finding that completely contradicts previous research (20, 35). To the best of our knowledge, long-term hemodialysis patients have elevated creatinine and urea levels, and the accumulation of uremic toxins results in a series of brain injuries, including neurotoxicity, neuroinflammation, blood–brain barrier injury, oxidative stress, microvascular changes, apoptosis, and brain metabolic dysfunction (45). However, among our patients, those with higher levels of creatinine and urea nitrogen had higher FC between the aMPFC and PCC. One possible explanation for this result is that the MPFC plays a regulatory role in the late stage of the disease, but this regulatory mechanism cannot fully compensate, and thus, patients still present a low degree of FC compared with HC controls. Further research should be carried out in the future to confirm this speculation.

### Disruption of Subsystems in Patients With ESRD

In addition to the predominantly disrupted cortical hubs in ESRD patients, we also found reduced FC in the dMPFC subsystem (dMPFC-TPJ) and MTL subsystem (pIPL-Rsp and pIPL-PHC), which may suggest further development of cognitive impairment in ESRD. The dMPFC subsystem is robustly activated for tasks that require mental state inference (24). There is strong connectivity and interaction between dMPFC and TPJ, constituting an important part of the “mentalizing network” (46), which may directly participate in the processing of emotions (47). The prefrontal cortex is found to be closely related to the production and regulation of emotions. Specifically, the dMPFC is recruited when involved in appraising the intentions and mental states of others, and in the expressing of negative emotion, and thus can affect the outcome of an individual's happiness (48). In contrast, the TPJ is recruited preferentially during the other-centered affective recognition process (49). These results indicate that the FC between the dMPFC and the TPJ may be the neural basis of the cognition process in emotion recognition and regulation. Moreover, depressive hemodialysis patients showed impaired FC in the amygdala-prefrontal-PCC-limbic circuits, as well as an abnormal interaction between depressive mood and cognitive control deficits (50, 51). Abnormal FC of the dMPFC subsystem was also found in patients with major depressive disorder (52). Therefore, the explanation for our findings is that lower connectivity between the dMPFC and the TPJ of the dMPFC subsystem might cause negative outcomes of the cognition process in emotion recognition and regulation, which may lead to the development of negative emotions, such as anxiety and depressive mood.

The MTL subsystem is preferentially activated when involved in episodic memory and thinking (24); thus, significant changes in the MTL subsystem may reflect cognitive changes in memory in ESRD patients. In particular, the Rsp is densely interconnected with several components of the hippocampal-parahippocampal memory system, which play a crucial role in the encoding of working memory, learning, and spatial processing (53). Additionally, the Rsp has connections with the posterior parietal cortex, which is responsible for working memory, visuo-spatial processing, and some related cognitive functions (54). According to previous studies, the bilateral IPL is reliably activated when performing working memory processes in both humans and animals (55). The PHC and Rsp mediated contextual associative memory as well as spatial learning (56), while the IPL was involved in the storage and expression of contextual details that support episodic memory in memory decisions as a temporary memory caching system (57). Moreover, a task-related fMRI study found similar FC (in the PCC, the bilateral IPL and the left PHC) abnormalities related to memory, consistent with our study (58). Thus, impaired FC among the pIPL, Rsp, and PHC in the MTL subsystems of the DMN in patients with ESRD may account for additional memory impairments to some degree.

## Limitations

Some limitations of the current study should be noted. First, patients with end-stage renal disease have severe metabolic imbalance, which is likely to have a substantial impact on cerebral vascular regulation. Rs-fMRI connectivity is not only driven by neurovascular but also merely vascular mechanisms. Although the current study tries to avoid the confounding effect, we did not have the ability to avoid the effect of these kinds of mechanisms, such as atherosclerosis. We selected hemodialysis patients to minimize the influence of different dialysis methods. In addition, the effects of physiological effects such as respiration and heart rate, especially the respiration-related “connectivity network” on functional connectivity, were not considered in this study. Second, 11 ROIs were selected from a previous study (24) based on data-driven approaches; other ROIs may also be worth studying. To avoid crossing results, these ROIs were located in the left hemisphere of the brain. It is not known whether the changes in brain function in ESRD patients have hemispheric dominance. However, a previous study have demonstrated that the left cerebral cortex has stronger functional connectivity with the same hemisphere (59). Third, we focused only on the DMN. Other cortical networks are equally important and may also be involved in the brain injury process in ESRD patients. Related research will be carried out in our future experiments. Fourth, the current study did not include cognitive testing, which would have allowed us to examine correlations with functional brain abnormalities. Five, these correlations between abnormal FC correlation and clinical variables did not pass stringent correction; thus, they were exploratory and need to be validated in a larger sample size. Next, the narrow selection criteria led to a small sample size, which limited the generalizability of our results. There were no significant group differences in connectivity between the vMPFC seed and any region, which might also be explained by the limited sample size. Therefore, further studies with larger groups of participants are needed to further confirm our findings. Finally, the study had a cross-sectional design; future work should include longitudinal studies to reflect changing patterns of DMN subsystem connectivity over the duration of dialysis.

## Conclusion

In conclusion, different subsystems of the DMN have inconsistent degrees of impairment in ESRD. The cortical hubs (PCC and aMPFC) are preferentially disrupted, and other subsystems may develop progressive impairment to a certain degree as the disease develops. Our findings provide novel insights into the underlying pathological mechanism of cognitive impairment in ESRD patients.

## Data Availability Statement

The raw data supporting the conclusions of this article will be made available by the authors, without undue reservation.

## Ethics Statement

The studies involving human participants were reviewed and approved by the Medical Research Ethics Committee of the Affiliated Hospital of Qingdao University, China. The patients/participants provided their written informed consent to participate in this study.

## Author Contributions

CM, Y-dR, and J-cF contributed to the conception and design of the study. CM, FT, M-gM, and H-wS collected the subjects, and Z-hL and CM contributed to the analysis of the resting-state fMRI data. CM wrote the first draft of the manuscript. Y-dR and J-cF performed critical revision of the manuscript for intellectual content. All authors contributed to manuscript revision and read and approved the submitted version.

## Conflict of Interest

The authors declare that the research was conducted in the absence of any commercial or financial relationships that could be construed as a potential conflict of interest.
